# Whole Exome Sequencing Reveals Compound Heterozygosity for Ethnically Distinct PEX7 Mutations Responsible for Rhizomelic Chondrodysplasia Punctata, Type 1

**DOI:** 10.1155/2015/454526

**Published:** 2015-10-26

**Authors:** Jessie C. Jacobsen, Emma Glamuzina, Juliet Taylor, Brendan Swan, Shona Handisides, Callum Wilson, Michael Fietz, Tessa van Dijk, Bart Appelhof, Rosamund Hill, Rosemary Marks, Donald R. Love, Stephen P. Robertson, Russell G. Snell, Klaus Lehnert

**Affiliations:** ^1^Centre for Brain Research and School of Biological Sciences, The University of Auckland, Auckland 1010, New Zealand; ^2^Adult and Paediatric National Metabolic Service, Auckland City Hospital, Auckland 1142, New Zealand; ^3^Genetic Health Service New Zealand, Auckland City Hospital, Auckland 1142, New Zealand; ^4^Department of Radiology, Auckland City Hospital, Auckland 1142, New Zealand; ^5^Department of Biochemical Genetics, SA Pathology, North Adelaide, SA 5006, Australia; ^6^Department of Diagnostic Genomics, PathWest, Nedlands, WA 6009, Australia; ^7^Department of Genome Analysis, Academic Medical Centre, 1105 Amsterdam, Netherlands; ^8^Department of Neurology, Auckland City Hospital, Auckland 1142, New Zealand; ^9^Developmental Paediatric Service, Starship Children's Health, Auckland 1142, New Zealand; ^10^Diagnostic Genetics, LabPLUS, Auckland City Hospital, Auckland 1142, New Zealand; ^11^Dunedin School of Medicine, University of Otago, Dunedin 9016, New Zealand

## Abstract

We describe two brothers who presented at birth with bone growth abnormalities, followed by development of increasingly severe intellectual and physical disability, growth restriction, epilepsy, and cerebellar and brain stem atrophy, but normal ocular phenotypes. Case 1 died at 19 years of age due to chronic respiratory illnesses without a unifying diagnosis. The brother remains alive but severely disabled at 19 years of age. Whole exome sequencing identified compound heterozygous stop mutations in the *peroxisome biogenesis factor 7* gene in both individuals. Mutations in this gene cause rhizomelic chondrodysplasia punctata, type 1 (RCDP1). One mutation, p.Arg232^*∗*^, has only been documented once before in a Japanese family, which is of interest given these two boys are of European descent. The other mutation, p.Leu292^*∗*^, is found in approximately 50% of RCDP1 patients. These are the first cases of RCDP1 that describe the coinheritance of the p.Arg232^*∗*^ and p.Leu292^*∗*^ mutations and demonstrate the utility of WES in cases with unclear diagnoses.

## 1. Introduction

Rhizomelic chondrodysplasia punctata, type 1 (RCDP1; OMIM 215100), resulting from mutations in* peroxisome biogenesis factor 7* (*PEX7*; OMIM 601757) is typically characterised by severe, prenatal defects in bone growth resulting in short stature, neurological impairment, and cataracts. Milder phenotypes with a growing genotypic and biochemical spectrum have been reported [1–3]. RCDP is estimated to affect approximately 1 per 100,000 children [1, 4], with type 1 accounting for greater than 90% of cases [3]. The* PEX7* gene encodes the receptor required for the targeting of peroxisomal proteins containing the peroxisome targeting signal 2, including the enzymes phytanoyl-CoA hydroxylase and alkyl-dihydroxyacetone phosphate synthase. These enzymes are involved in peroxisomal *α*-oxidation of phytanic acid and the synthesis of plasmalogen ether lipids, respectively. Interruption of these processes results in the key biochemical hallmarks of RCDP1, elevated plasma phytanic acid and low erythrocyte plasmalogen levels, which occur in the presence of normal plasma very long chain fatty acid (VLCFA) levels (reviewed by Wanders [5]). We describe a sib-pair with variable phytanic acid levels coupled with normal levels of VLCFA who presented with global developmental delay, mild epiphyseal dysplasia, epilepsy, poor growth, subtle dysmorphism, and cerebellar and brain stem atrophy. The two affected individuals are the only live-born children (seven miscarriages) to unrelated parents of European descent. Using whole exome sequencing we identified two stop mutations in the* PEX7* gene, and a subsequent diagnosis of RCDP1 was made.

## 2. Case 1

Case 1 was born at term after a pregnancy complicated by deficiency in amniotic fluid. He had low muscle tone and required oxygen and bag-mask resuscitation immediately after birth. He breathed normally at 3 minutes (Apgar [6] scores of 3 and 9 at one and five minutes after birth, resp.). Birth weight (3210 g) and head circumference (34 cm) were on the 25th percentile. He was noted initially to have micrognathia and contractures of the hands and feet but fed well and was discharged at day 3. By 6 months, developmental delay across all milestones was apparent and growth was well below the 3rd percentile (6 kg at 7 months). At 3 years of age, growth defects at the ends of his long bones (epiphyseal dysplasia with flared metaphyses and small epiphyseal ossification centres) were reported. Radiographs at 12 years of age showed punctate patellar ossification centres and marked metaphyseal splaying (Supplementary Figure 1 in Supplementary Material available online at http://dx.doi.org/10.1155/2015/454526). Development remained markedly delayed with treatment-refractory epilepsy resulting in regression. Growth remained poor with a weight of 10 kg at 6 years of age (≪ 3rd percentile) despite supplemental feeding, and vision assessments were normal (last performed at 10 years). A peroxisomal disorder was initially considered, and elevated phytanic acid level with normal VLCFAs was reported at 6 years of age. However, repeat phytanic acid level was normal, and thus a peroxisomal disorder was thought to have been excluded (Supplementary Table 1). Brain magnetic resonance imaging (MRI) at 10 years of age revealed delayed myelination and atrophy of the brain stem and cerebellum (Supplementary Figure 2). He developed progressive scoliosis and recurrent chest infections and died at 19 years of age with no unifying diagnosis. Postmortem examination confirmed an abnormal hindbrain and brainstem.

## 3. Case 2

Case 2 was born at 35 weeks' gestation by elective caesarean section due to poor fetal growth and decreasing liquor from 32 weeks. Apgar scores were within normal range (8^1^ and 9^5^). He had flexion contractures of the knees and required respiratory support for 24 hours. Punctate calcifications of his patellae, proximal femurs, and humerus (head and medial epicondyles) were evident, but there were no reports of shortened limbs. He later developed evidence of epiphyseal dysplasia with metaphyseal flaring (Supplementary Figure 1). He had slightly more prominent facial dysmorphism and was less alert and interactive but otherwise followed a similar developmental and growth trajectory to his brother. At 17 years of age he weighed 19.6 kg. He had severe intellectual and physical impairment with increasing encephalopathy and treatment-refractory epilepsy which started at 6 years of age. He was dependant for all activities of daily living and was largely noncommunicative. There was no clear evidence of multisystem disease and no cataracts. Brain MRI at 6 years of age was similar to his brother and a repeat scan at 17 years of age revealed moderate atrophy of the cerebellum (Supplementary Figure 2). A neurometabolic workup was completed. The only abnormalities were elevated neopterin and low biogenic monoamine metabolites, which were thought to be secondary to brain disease. Phytanic acid was elevated and VLCFAs were reported as normal (Supplementary Table 1).

## 4. Exome Sequencing

To identify the causal mutations underlying this sib-pair, we performed whole exome sequencing of both brothers. The study was approved by the New Zealand Northern B Health and Disability Ethics Committee (12/NTB/59), and parents provided written informed consent. We obtained average coverage of 71 and 74, respectively, across the targeted regions and discovered 46,794 high-quality variants with concordant genotypes in the sib-pair. The 19,439 homozygous variants were filtered to remove genotypes observed more than once in 123 control New Zealand exomes and variants with alleles frequently observed in Europeans (Supplementary Table 2). Only one of the remaining 114 homozygous variants was potentially functional (*RTKN2*) but was not located in a gene related to cerebellar ataxia or atrophy.

**Figure 1:**
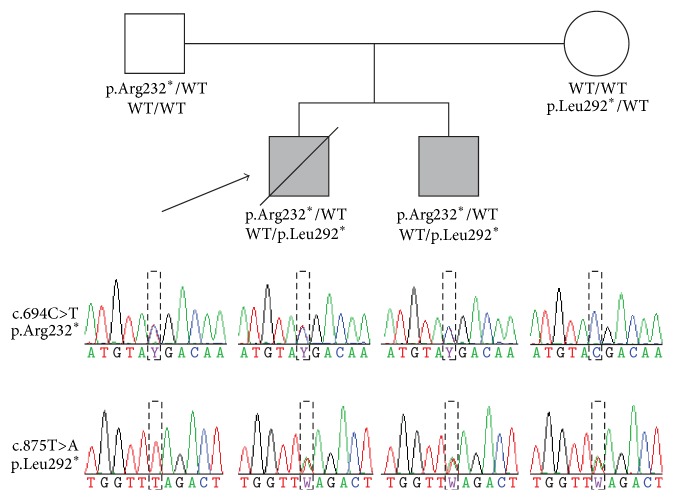
Family pedigree and transmission of the c.694C>T and c.875T>A mutations in the* PEX7* gene. The arrow identifies case 1. Protein genotypes are indicated immediately below each family member's pedigree symbol, and Sanger sequencing electropherograms for both loci are shown below the corresponding family member in the lower part of the figure. The couple's seven miscarriages are not depicted. WT, wild-type allele.

Of the 27,355 heterozygous variants identified in both siblings, 1,447 were rare or absent in the surveyed European population or control exomes; of these, 238 variants were potentially functional. Five genes carried more than one rare and functional variant and met our minimum criteria for potentially compound recessive inheritance (Supplementary Table 2). Nonsynonymous mutations in* CHTF18*,* FSIP2*, and* MYOM2* were excluded from further consideration based on the genes' known biological functions or disease associations. Two nonsynonymous mutations were identified in* CACNA1H* (RefSeq accession NG_012647.1; OMIM 607904), which is a susceptibility gene for childhood absence epilepsy (OMIM 611942). Two stop-gain variants, c.694C>T, p.Arg232^*∗*^ (rs121909153) and c.875T > A, p.Leu292^*∗*^ (rs1805137), in the* PEX7* gene (RefSeq accession NG_008462.1) were the most functionally significant mutations and were validated by PCR and Sanger sequencing in the affected children and their unaffected parents, confirming compound heterozygous inheritance (Figure 1). Both mutations are extremely rare and have been previously identified as pathogenic, causing RCDP1. The p.Leu292^*∗*^ mutation is estimated to account for >50% of RCDP1 cases [1, 2, 7]. The p.Arg232^*∗*^ mutation has only been published in a single consanguineous Japanese parent-child trio [8, 9] and reported in a heterozygous carrier from South Asia [10]. This is the first time, to our knowledge, it has been identified in individuals of European ancestry. Following genetic diagnosis (at 18 years of age) erythrocyte phospholipids (plasmalogens) were also found to be markedly reduced, which is consistent with a diagnosis of RCDP1 (Supplementary Table 1).

## 5. Discussion

Whole exome sequencing identified two compound heterozygous stop-gain mutations in the* PEX7* gene in two New Zealand children of European descent who primarily presented with global developmental delay, mild bone growth defects, epilepsy, poor growth, subtle facial dysmorphism, and cerebellar and brain stem atrophy. Cerebellar atrophy is a well-documented phenomenon in RCDP1 and brainstem pathology has been previously reported in peroxisomal disorders [11–13]. Ichthyotic skin, cleft palate, and congenital heart disease were not observed in either case. Proximal limb shortening and cataracts were not noted in the children, and their neurological deterioration and poor growth were severe but attenuated compared to classical RCDP1. Interestingly, bilateral cataracts were also not observed in the only other case reported with an Arg232^*∗*^ mutation [9], broadening the phenotypic spectrum of RCDP1 and emphasising the importance of diagnosis by next generation sequencing. The* PEX7* mutations described here have been previously reported, but not in this combination. One of the mutations, p.Leu292^*∗*^, accounts for ~50% of RCDP1 cases and is due to a founder effect in Northern European Caucasian populations [1]. The second mutation, p.Arg232^*∗*^, has only been published in a single family of Japanese descent, and a single heterozygous individual from South Asia has been reported in the ExAc database [10] (global allele frequency = 8.23 × 10^−6^). The presented sib-pair shows that the mutation is not confined to Asian populations and suggests it should be screened in European cases.

In neonates suspected of RCDP1, biochemical diagnosis of the disorder is traditionally performed by the detection of reduced erythrocyte plasmalogen levels; elevated phytanic acid levels are usually only observed after 6–12 months of age, due to phytanic acid being derived from the diet. However, in older patients (greater than 12 months of age), plasma phytanic acid analysis is often used as a primary biochemical tool for the diagnosis of RCDP1. In this study, analysis of the older sibling at 6 years of age yielded an elevated and then normal phytanic acid level. Fluctuating phytanic acid levels were also obtained in case 2. These results, coupled with normal levels of VLCFA, and more subtle clinical features than expected resulted in the erroneous exclusion of a peroxisomal disorder despite reviews from multiple specialists. Although earlier studies have shown the presence of normal phytanic acid levels in children with milder presentations of RCDP1 [11, 14], the relatively severe clinical presentation of both siblings, together with the presence of two previously described nonsense variants in the* PEX7* gene, makes the observed oscillating phytanic acid levels quite unexpected. This result emphasises the biochemical spectrum of RCDP1 and indicates that, in cases suspected of RCDP1, erythrocyte plasmalogen analysis should always be performed in parallel with phytanic acid testing.

Interestingly, the children also harbour two previously identified variants (rs57552791 and rs58173258) in the* CACNA1H* gene (inherited in a recessive compound heterozygous fashion), which is a susceptibility gene for childhood absence epilepsy. The rs58173258 variant (p.A876T) has been found to segregate with disease status in one family with epilepsy studied by Heron et al. [15]; however, the phenotypes within the family were diverse. When studied* in vitro*, the variant was found to alter channel activity resulting in an increase in channel function [15]. Therefore, it is possible that these two single nucleotide variants contribute to the epilepsy phenotype observed in these two children, although their seizures do not appear to be more pronounced than those observed in other RCDP1 patients and their MRI findings were typical of those with severe RCDP1 [11]. Given that the majority of RCDP1 patients develop seizures, further research would need to be conducted to deduce the underlying role of these* CACNA1H* variants.

The rapid developments and decreasing cost of exome and whole genome sequencing are making diagnosis of phenotypically diverse conditions such as RCDP1 easier. This is exemplified by the sib-pair presented here where diagnosis through whole exome sequencing provided the scaffold for interpretation of their clinical histories and biochemical results, which did not entirely fit the classical description of RCDP1. This methodology allowed the unifying diagnosis to be made 26 years after birth of case 1, vouching for the use of next generation sequencing for the diagnosis of severe neurodevelopmental disabilities.

## Supplementary Material

Supplementary materials include radiographs, magnetic resonance images and biochemical measures for the cases. Also included are methodological details and variant filtering results from the whole exome sequencing analysis.
