# Supplementary material for: Whole Exome Sequencing Reveals Compound Heterozygosity for Ethnically Distinct PEX7 Mutations Responsible for Rhizomelic Chondrodysplasia Punctata, Type 1
Source: Case Rep Genet. 2015 Oct 26;2015:454526. doi: 10.1155/2015/454526 (PMC4637447; doi:10.1155/2015/454526)
Supplement: Supplementary file 1 — Supplementary materials include radiographs, magnetic resonance images and biochemical measures for the cases. Also included are methodological details and variant filtering results from the whole exome sequencing analysis. [file 454526.f1.pdf]

Supplementary Figures

Supplementary Figure 1: Case 1 and 2 radiographs

A) Case 1, 12 years of age: Plain radiograph of the left knee demonstrating metaphyseal splaying and punctate patellar ossification centre. B) Case 1, 14 years: Plain radiograph of left humerus demonstrating both proximal and distal metaphyseal splaying. C) Case 2, 12 years: Plain radiograph of the pelvis demonstrating marked flattening of the femoral heads with epiphyseal dysplasia and metaphyseal flaring. D) Case 2, 12 years: Plain radiograph of the chest demonstrating stippled epiphysis of the left shoulder and bilateral metaphyseal splaying.

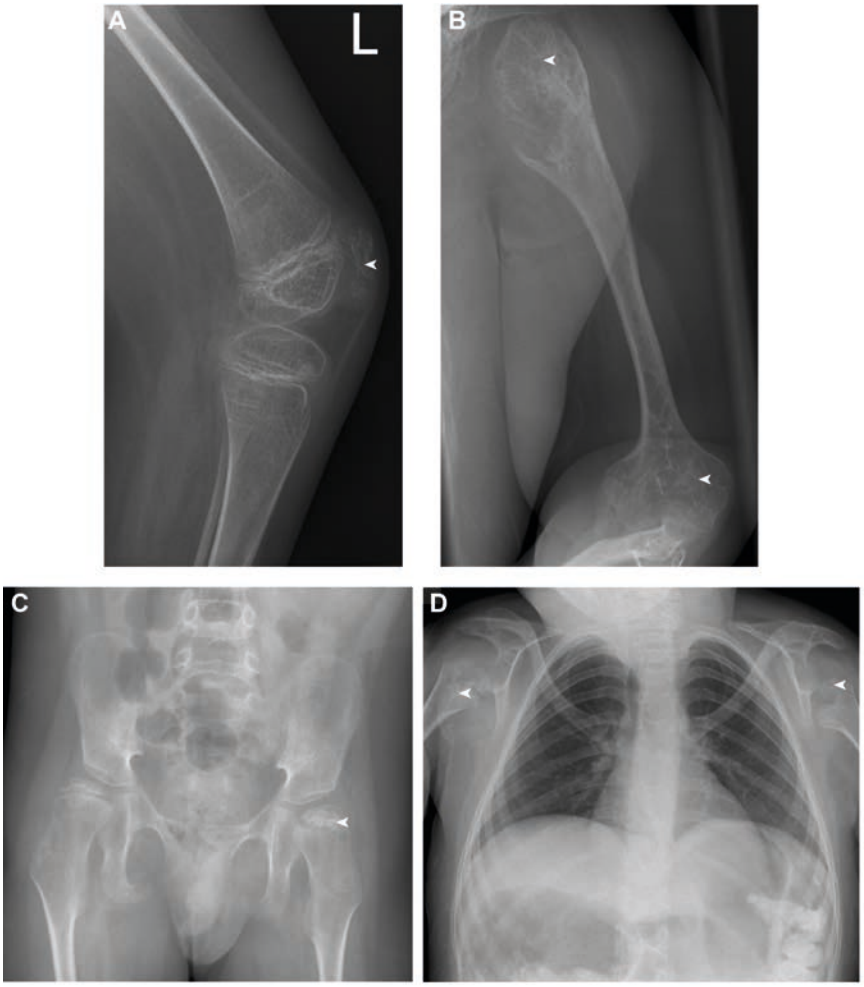

Supplementary Figure 2: Case 1 and 2 magnetic resonance imaging

A) Case 1, 10 years: Sagittal T1 MRI demonstrating small cerebellar vermis and mildly hypoplastic pons. B) Case 2, 17 years: Coronal T2 MRI demonstrating hypoplastic cerebellum with prominent folia and increased CSF space in the posterior fossa. C) Case 2, 17 years: Sagittal T2 MRI demonstrating cerebellar atrophy and mild atrophy of pons.

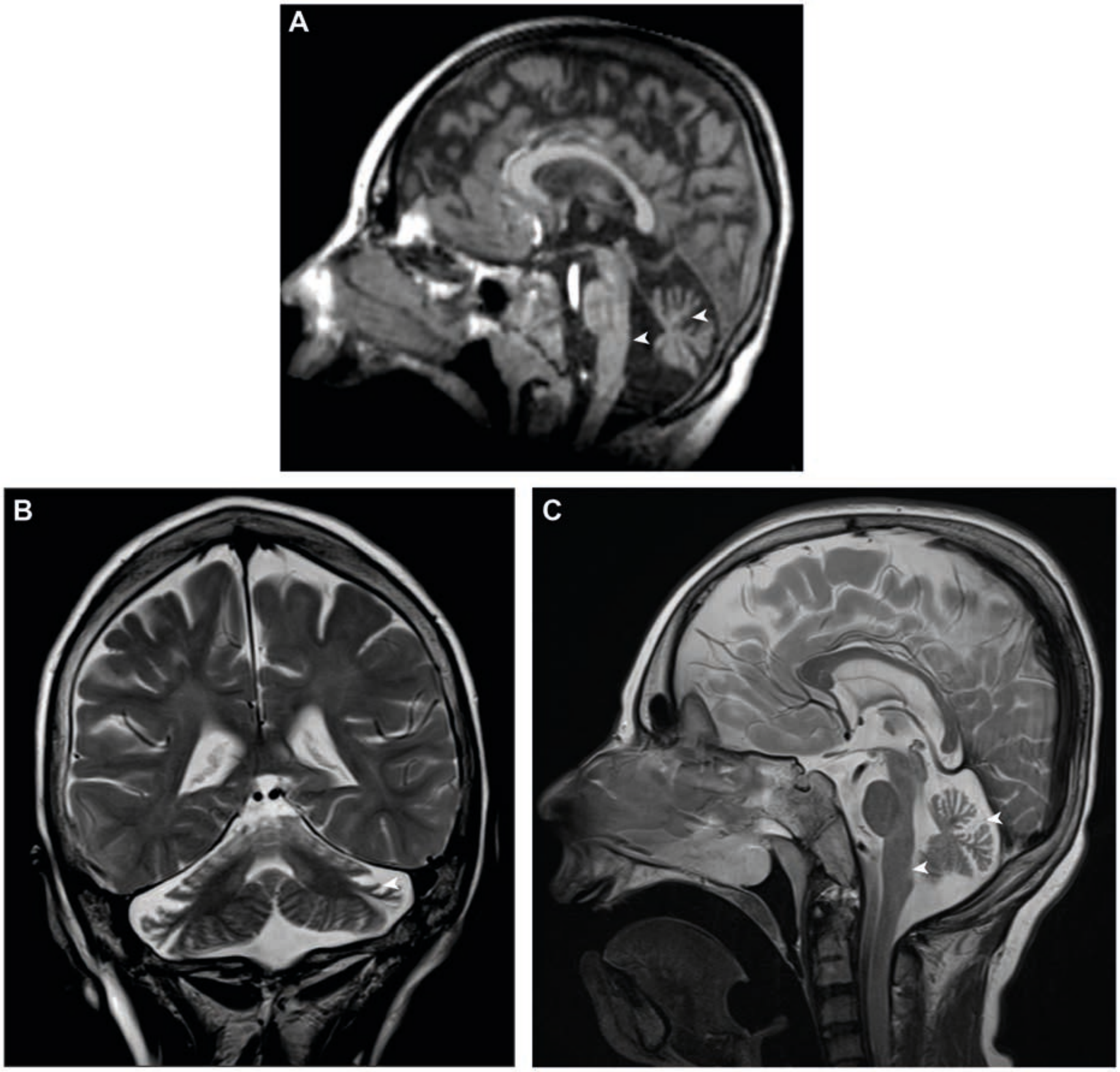

Supplementary Tables

Supplementary Table 1: Biochemical investigations

Abnormal biochemical investigations performed on case 1 at 6 years after being seen in Gottingen, Germany and case 2 at 6 months, 17 years and 18 years. These results demonstrate the fluctuating levels of phytanic acid and show the importance of testing plasmalogen levels to exclude or confirm RCDP1. Abnormal results are in bold. Normal ranges are in brackets and differ depending on the lab and methodology.

**Investigation**	**Case 1 (6 years)**	**Case 2 (6 months)**	**Case 2 (17 years)**	**Case 2 (18 years)**
Plasma Hexacosanoate C26:0	Not done	Not done	**0.29 µmol/L** (0.33-1.13)	Not done
Plasma C24:0/C22:0 ratio	Not done	Not done	**0.006** (0.007-0.023)	**0.512** (0.55-1.115)
Plasma C26:0/C22:0 ratio	Reported as 'normal' - data unavailable	0.087 (<0.15)	0.56 (0.60-1.02)	0.008 (<0.035)
Plasma Phytanic acid (3,7,11,15-tetramethyl hexadecanoic acid)	**60 ug/ml** (<30)<br>Repeat: 27 ug/ml	Not done	**43.27 µmol/L** (0.82-11.09)<br>Repeat: Reported as non-specifically abnormal in a pattern not suggestive of an inborn error of metabolism	**1.2 mg/100mL** (<0.7)
Plasma Pristanic acid (2,6,10,14-tetramethylpentadecanoic acid)	Not done	Not done	**Not detected**	Not done
Erythrocyte C16:0 plasmalogens	Not done	Not done	Not done	**11 µg/g Hb** (140-300)
Erythrocyte C18:0 plasmalogens	Not done	Not done	Not done	**27 µg/g Hb** (265-475)
Skin fibroblast catalase activity	Not done	10.2 µmol/min/mg (4.5-20.0)	Not done	Not done
Cerebrospinal fluid monoamine metabolites	Not done	Not done	Neopterin **91.7 nmol/L** (6.0-30.0)<br>Biopterin 33.3 nmol/L (25-45)<br>5-hydroxyindolacetic acid **0.26 µmol/L** (0.06-0.19)<br>Homovanillic acid 0.30 µmol/L (0.09-0.37)	Not done

Supplementary Table 2: Variant filtering

	homoz rec model	homoz rec model	comp rec model	comp rec model
	Variants	Genes	Variants	Genes
Variants with concordant genotype	19,439	7,703	27,355	8,522
Alternate allele frequency ≤ 0.2 in ESP, 1000G, HapMap, or ExAc Europeans	4,275	2,809	6,794	3,926
Genotype frequency ≤ 0.02 (homoz) or ≤ 0.2 (comp) in control exomes	114	108	1,447	1,254
Potentially functional	1	2	236	232
Located in genes with > 1 rare functional variant	n/a	n/a	8	4
Located in known cerebellar ataxia/atrophy gene	0	0	2	1

Supplementary Methods

Whole exome sequencing

DNA was extracted from whole blood and exome capture was performed using the Agilent SureSelect Human All Exon V5 kit, followed by paired-end sequencing (Illumina HiSeq 2000/2500). 58 million reads were obtained for each sibling and mapped to GRCh37.p13 reference genome using Burrows-Wheeler Aligner (v0.7.10[17]), followed by identification of PCR and optical duplicates with Picard (v1.96, picard.sourceforge.net). Reads were realigned around indels and base quality scores were recalibrated with the GenomeAnalysis Toolkit (GATK v3.3-0[18]). Variants in the targeted regions and flanking 50 nucleotides were identified using the GATK HaplotypeCaller algorithm after supplementing exome alignments from 124 unrelated European control individuals to support variant discovery and empower variant quality score recalibration[19]. Variants discovered in each patient were annotated using SeattleSeq Annotation 138[20], snpEff (v3.5[21]), and custom scripts. Variants were considered potentially functional if they resulted in gain or loss of a stop or start codon, caused a frameshift, codon deletion or insertion, non-synonymous amino acid substitution, located in the 5'- or 3'-untranslated regions or were within 5 nucleotides of a splice consensus motif. Alleles were considered 'frequent' if their frequencies exceeded 0.2 in the European subsets of the HapMap, 1000 Genomes, NHLBI Exome Sequencing Project, or Exome Aggregation Consortium cohorts[11], or if the genotype frequency in 123 New Zealand control exomes exceeded 0.2 (for heterozygous variants) or 0.02 (for homozygous variants).

Mutation validation/PCR and Sanger sequencing

PCR amplification of each of the *PEX7* variants was performed using standard conditions with the following primers (PEX7 F1 5’ TCAAAAATGACTCCTTGGTTCA 3’ and PEX7 R1 5’ TTTTTGCCTAATTTTAGCTTTCA 3’; PEX7 F2 5’ GGCTGAGCCTGTTTGGATAA 3’ and PEX7 R2 5’ TCCTGGGAAGGTTCCTTTTT 3’). PCR products were Sanger sequenced using the Applied Biosystems 3130XL Genetic Analyser at the Centre for Genomics and Proteomics at The University of Auckland. Both mutations were independently validated using whole exome sequencing at the Academic Medical Center (AMC) in the Netherlands.
